# Replicative Bypass of Abasic Site in *Escherichia coli* and Human Cells: Similarities and Differences

**DOI:** 10.1371/journal.pone.0107915

**Published:** 2014-09-16

**Authors:** Savithri Weerasooriya, Vijay P. Jasti, Ashis K. Basu

**Affiliations:** Department of Chemistry, University of Connecticut, Storrs, Connecticut, United States of America; University of Massachusetts Medical School, United States of America

## Abstract

Abasic [apurinic/apyrimidinic (AP)] sites are the most common DNA damages, opposite which dAMP is frequently inserted (‘A-rule’) in *Escherichia coli*. Nucleotide insertion opposite the AP-site in eukaryotic cells depends on the assay system and the type of cells. Accordingly, a ‘C-rule’, ‘A-rule’, or the lack of specificity has been reported. DNA sequence context also modulates nucleotide insertion opposite AP-site. Herein, we have compared replication of tetrahydrofuran (Z), a stable analog of AP-site, in *E. coli* and human embryonic kidney 293T cells in two different sequences. The efficiency of translesion synthesis or viability of the AP-site construct in *E. coli* was less than 1%, but it was 7- to 8-fold higher in the GZGTC sequence than in the GTGZC sequence. The difference in viability increased even more in pol V-deficient strains. Targeted one-base deletions occurred in 63% frequency in the GZG and 68% frequency in GZC sequence, which dropped to 49% and 21%, respectively, upon induction of SOS. The full-length products with SOS primarily involved dAMP insertion opposite the AP-site, which occurred in 49% and 71% frequency, respectively, in the GZG and GZC sequence. dAMP insertion, largely carried out by pol V, was more efficient when the AP-site was a stronger replication block. In contrast to these results in *E. coli*, viability was 2 to 3 orders of magnitude higher in human cells, and the ‘A-rule’ was more rigidly followed. The AP-site in the GZG and GZC sequences gave 76% and 89%, respectively, Z→T substitutions. In human cells, targeted one-base deletion was undetectable, and dTMP>dCMP were the next preferred nucleotides inserted opposite Z. siRNA knockdown of Rev1 or pol ζ established that both these polymerases are vital for AP-site bypass, as demonstrated by 36–67% reduction in bypass efficiency. However, neither polymerase was indispensable, suggesting roles of additional DNA polymerases in AP-site bypass in human cells.

## Introduction

Abasic sites, also called apurinic or apyrimidinic sites (AP-sites), are one of the most common lesions in DNA [1]. They occur both spontaneously and following chemical modification of DNA [2]–[6]. AP-sites are non-coding and they strongly block DNA replication by replicative DNA polymerases (pols). A specialized class of pols, known as the transelsion synthesis (TLS) enzymes, however, can bypass DNA damages, including AP-sites [7]. But AP-sites lack the information necessary to identify the correct base. So, when a nucleotide is incorporated opposite AP-site, it frequently results in a mutation [8], [9].

In *Escherichia coli* cells, AP-site bypass is largely SOS-dependent [8] and dAMP is most commonly inserted opposite it, which led to the so-called ‘A-rule’ [10]. Site-specific studies in *E. coli* showed that the preferential dAMP insertion occurs with SOS [11]. In *Saccharomyces cerevisiae*, dCMP is inserted (‘C-rule) when the AP-site is located in the single-stranded gap of a gapped duplex plasmid [12], but in duplex DNA dAMP is inserted preferentially opposite the AP-site in both the leading and lagging strand [13]. In simian kidney cells, some studies suggest a lack of specificity in nucleotide insertion opposite AP-sites [14]–[16]. In human cells, by contrast, in the single-stranded gap region of a gapped duplex plasmid, the majority of the bypass involved insertion of dAMP opposite AP-sites [17]. The natural AP-site remains in ring-chain equilibrium of the cyclic hemiacetal with an open chain aldehyde form, which is highly labile to heat and alkaline pH [18]. Because of its instability, a tetrahydrofuran (Z), as a stable model of the cyclic form of abasic site, has been used in many studies. A comparison of DNA replication past the natural AP-site and Z, the stable analog, in *E. coli* showed that the biological effects of these two lesions are similar in SOS-induced *E. coli*, but their replication characteristics are distinct in uninduced cells [19].

The discovery of the Y-family polymerases [20], [21] that can efficiently bypass DNA lesions generated interest in investigating the kinetics of TLS of these enzymes on AP-site templates. Pol V (UmuD′_2_C complex) can bypass AP-site efficiently *in vitro* incorporating dAMP preferentially opposite it, whereas pol III and pol IV failed to bypass it on the same time scale [22]. But pol IV incorporates a nucleotide opposite the AP-site and so another polymerase may carry out the extension step [22]. Both yeast and human pol η can efficiently bypass AP-sites and both prefer to insert purine nucleotides [13], [23]. The yeast enzyme, in addition, causes high frequency of −1 and −2 frameshifts [24]. Dpo4 and human pol κ also bypass AP-sites, although with significantly reduced efficiency [25], [26]. For human pol κ, however, accessory proteins such as PCNA, RFC, and RPA increase efficiency by more than an order of magnitude [26]. Human pol ι also bypasses AP-sites inserting either dGTP or dTTP with 10-fold reduced efficiency [27]. Pol δ and REV1, in the presence of pol ζ, can bypass AP-sites [28], [29]. It was suggested that pol δ inserts dAMP preferentially opposite the AP-site, and pol ζ subsequently extends from the inserted nucleotide [28], but arguments against this model have been presented [29].

Despite the wealth of data from the *in vitro* experiments described above, the mechanism of AP-site bypass in a cell remains unclear. Additional complications arise from the fact that nucleotide insertion opposite the AP-site is greatly dependent on the DNA sequence context and the type of cells. Consequently, evaluation of data from different laboratories using different sequence contexts and different types of cells could be challenging. In the current study, we compared TLS of Z, the stable analog of abasic site, in *E. coli* and human embryonic kidney (HEK) 293T cells in two different sequence contexts. We report herein that sequence context plays a major role in bypassing AP-site and that the ‘A-rule’ is followed in human cells. We also find that in *E. coli*, pol V is a major but not the only pol that follows ‘A-rule,’ whereas in human cells, Rev1 and pol ζ play important roles in AP-site bypass.

## Materials and Methods

### Materials

[γ-^32^P]ATP was from Du Pont New England Nuclear (Boston, MA). T4 DNA ligase and T4 polynucleotide kinase were obtained from New England Biolabs (Beverly, MA). *Escherichia coli* strain DH10B was purchased from Invitrogen (Carlsbad, CA). HEK 293T cells were obtained from ATCC (Manassas, VA). Single-stranded phagemid pMS2 DNA was prepared from *E. coli* JM109 with the aid of the helper phage M13K07 (NEB, Beverly, MA) as reported by Moriya [30].

### Lesion Containing Oligonucleotides

Z containing and control dodecamers of the sequences 5′-TGCAGZGTCAGC-3′, 5′-TGCAGTGZCAGC-3′, and 5′-TGCAGTGTCAGC-3′ were synthesized by the Midland Certified Reagent Company, Inc (Midland, TX). The oligonucleotides were purified by C18 reverse phase HPLC followed by denaturing polyacrylamide gel electrophoresis. Mass spectrometric analysis by MALDI-TOF and/or ESI-MS verified the molecular weight of the oligonucleotides. Additionally, MS and polyacrylamide gel electrophoresis analyses indicated that the Z containing oligonucleotides were more than 99% pure.

### Construction of M13 genome containing a single abasic site and replication in *E. coli*

Construction of the modified and control M13 genome involved digestion of M13mp7L2 single-stranded DNA with *Eco*RI, annealing a 50-mer scaffold, and ligation of the Z-containing and control 12-mer, which followed the protocol described earlier in detail [31]. The efficiency of ligation was ∼40% for both the control and modified 12-mer. Removal of the scaffold 50-mer and transfection of the genomes in *E. coli* also followed published procedure. Analysis of progeny phage was carried out by oligonucleotide hybridization followed by DNA sequencing.

### Construction of pMS2 plasmid containing a single abasic site and replication in HEK 293T cells

The single-stranded pMS2 shuttle vector DNA, containing a hairpin region, was digested with *Eco*RV and a 58-mer scaffold oligonucleotide was annealed overnight at 9°C to form the gapped DNA, as reported [32]. The control and lesion containing 12-mers were phosphorylated with T4 polynucleotide kinase, hybridized to the gapped pMS2 DNA, and ligated overnight at 16°C [32]. Unligated oligonucleotides were removed by passing through Centricon-100 and the DNA was precipitated with ethanol. The scaffold removal followed the same protocol as described [31], [32]. The final construct was dissolved in 1 mM Tris-HCl-0.1 mM EDTA, pH 8, and a portion was subjected to electrophoresis on 1% agarose gel in order to assess the amounts of circular DNA. Based on the proportion of circular DNA on the agarose gel, the ligation efficiency for each Z-containing dodecamer and the control was estimated to be ∼50%.

### SOS induction and transformation in *E. coli*

*E. coli* cells were grown in 100 mL cultures to 1×10^8^ cells/mL and then harvested by centrifugation at 5000 g for 15 min at 0°C. This procedure was repeated twice except the cells were resuspended in 40 mL ice-cold deionized water. The bacterial pellet was resuspended in 1 mL of glycerol/water (10% v/v) and kept on ice until further use. For SOS response, the following additional steps were introduced after the first centrifugation. The cells were resuspended in 50 mL 10 mM MgSO_4_ and treated with 50 J/m^2^ of UV light (254 nm) in 25 mL aliquots in 150×50 mm plastic petri dishes. The cultures were incubated in Luria broth at 37°C for 40 min in order to express SOS functions maximally. Following SOS induction, these cells were centrifuged, deionized, and resuspended in glycerol/water in a similar manner as described earlier except all manipulations were carried out in subdued light. For each transformation, 40 µL of the cell suspension was mixed with 1 µL (100 ng) DNA solution and transferred to the bottom of a cold Bio-Rad Gene-Pulser cuvette. Electroporation of cells was carried out in a Bio-Rad Gene-Pulser apparatus at 25 µF and 2.5 kV with the pulse controller set at 200 Ω. Immediately after electroporation, 1 ml SOC medium was added. The cells were incubated for 1 h at 37°C to allow for phage replication and subsequently centrifuged at 15000× g (5 min) to isolate the phage-containing supernatant. Oligonucleotide probes containing the complementary 15-mer sequence were used for analysis. Two 14-mer left and right probes were used to select phages containing the correct insert, and transformants that did not hybridize with both the left and right probes were omitted. Any transformant that hybridized with the left and right probes but failed to hybridize with the 15-mer wild-type probe were subjected to DNA sequence analysis. Lesion bypass efficiency was calculated by comparing the transformation efficiency of the AP-site-containing construct with that of the control, whereas mutation frequency (MF) was calculated on the basis of hybridization and sequence analysis.

### Replication and analysis in human embryonic kidney 293T cells

The HEK 293T cells were maintained in Dulbecco's modified Eagle's medium supplemented with 4 mM L-glutamine, and adjusted to contain 1.5 g/L sodium bicarbonate, 4.5 g/L glucose, and 10% fetal bovine serum. The cells were grown to ∼90% confluency and transfected with 50 ng of each construct using 6 µL of Lipofectamine cationic lipid reagent (Invitrogen, Carlsbad, CA). Subsequent to transfection with modified or unmodified pMS2, the cells were allowed to grow at 37°C in 5% CO_2_ for 2 days and the plasmid DNA was collected and purified by the method of Hirt [33]. It was used to transform *E. coli* DH10B and the analysis was performed similarly as described above.

### TLS assay in HEK 293T cells

The AP-site-containing or control pMS2 construct was mixed with equal amount of a single-stranded pMS2 DNA containing the DNA sequence 5′-GTCCGTGTTTGT-3′ instead of 5′-TGCAGTGTCAGC-3′ in the region where Z was placed. The mixed DNA was used to transfect HEK293T cells and processed as described above. Oligonucleotide probes for the complementary sequences for both the wild type and the mutant plasmid were used to analyze the progeny. The mutant DNA was used as an internal control and it gave approximately equal number of progeny as the control construct.

### Mutational analyses of TLS products from human cells with either Rev1 or pol ζ knockdown

Prior to transfection of the control and Z-containing vectors, synthetic siRNA duplexes were transfected into HEK293T cells using Lipofectamine. HEK293T cells were plated in 6-well plates at 50% confluence. After 24 h incubation, they were transfected with 100 pmoles of siRNA duplex mixed with Lipofectamine, diluted in Opti-MEM (Gibco), per well. One day before transfection of the plasmid, cells were seeded in 24-well plates at 70% confluence. Cells were then co-transfected with another aliquot of siRNA and either control plasmid or Z-containing plasmid at a ratio of 2∶1. After 24 h incubation, progeny plasmids were isolated as described earlier.

## Results

### Construction of the AP-site containing vector and its replication

**Figure 1:**
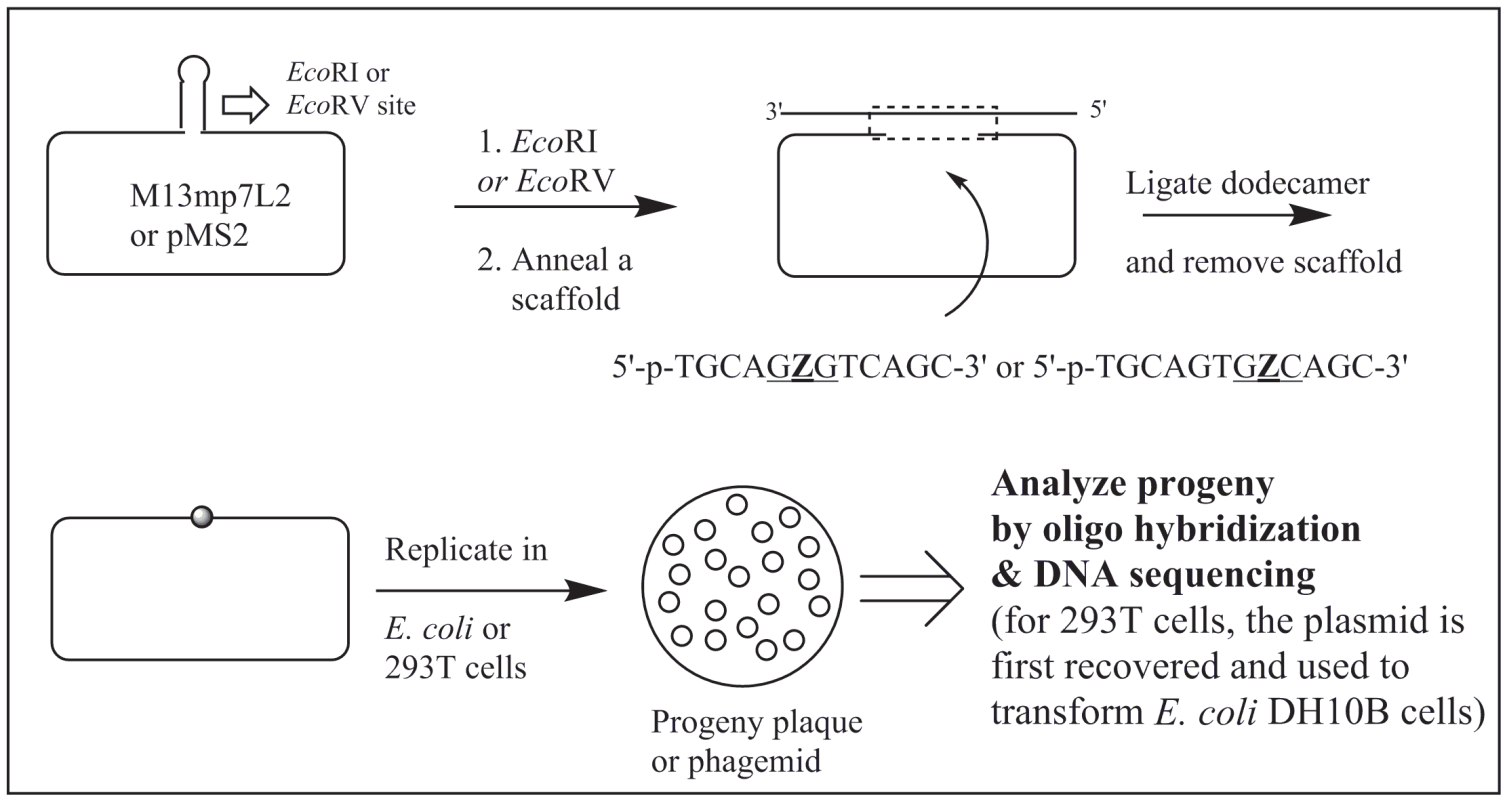
A general scheme for construction of the lesion-containing vector, its replication in *E. coli* or HEK 293T cells, and analysis of the progeny.

We used the M13mp7L2 vector [34] to investigate TLS of Z in *E. coli* cells, whereas the same in HEK 293T was carried out using the pMS2 shuttle vector plasmid containing the origins of replication for f1, ColE1, and SV40, and the genes for neo and amp resistance [35], [36]. Both single-stranded vectors contain a hairpin region that, upon digestion with the appropriate restriction enzyme followed by scaffolding with an oligonucleotide, generates the desired gapped DNA. The Z containing 12-mer is ligated to this gap and the scaffold is removed before replication. The strategy for construction and mutational assay is shown in Figure 1.

### TLS of Z in *E. coli* Cells

**Figure 2:**
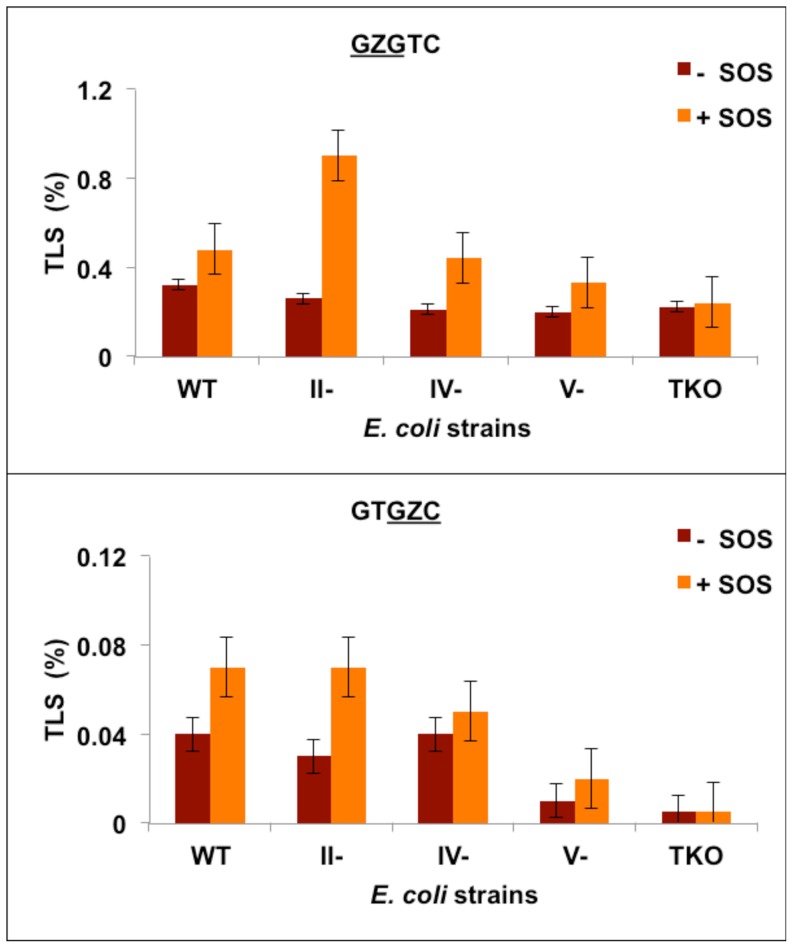
TLS frequencies for GZGTC and GTGZC constructs in wild type and pol II-, pol IV-, pol V-, and triple-knockout *E. coli* strains without (−) and with (+) SOS.

Magnitude of TLS or viability was determined by comparing the transformation efficiencies of Z-containing and control constructs. As shown in Figure 2 and Table S1, Z is a highly toxic lesion, and in the absence of SOS-induction, viability was only 0.04% and 0.3%, respectively, in the GZC and GZG sequence. With SOS, viability increased by 50–75%. The increase in viability with SOS is lower than what others have reported with either natural abasic site or Z [11], [19], [24]. Since SOS induction was considered optimal by parallel replication of other lesions [37], [38], we suspect that the sequence context may have played a role in this relatively modest enhancement in viability. What we found noteworthy, however, is that the viability of the GZC construct was 7- to 8-fold lower than that of the GZG sequence, in both uninduced and SOS-induced *E. coli* (Figure 2 and Table S1), which suggests that the GZC sequence poses a stronger replication block than the GZG sequence. Viability followed a similar trend in pol II- or pol IV-deficient cells, although it was increased nearly 3-fold with SOS in the GZG sequence. For the GZC sequence, viability was 3- to 4-fold lower in the pol V-deficient strain, whereas it was 8- to 14-fold lower in the triple knockout (TKO) strain that lacks pol II, pol IV, and pol V (Figure 2). But such pronounced decrease in viability was not detected in the GZG sequence, so that the viability in GZC sequence relative to GZG sequence was reduced by at least 15-fold and 48-fold, respectively, in pol V-deficient and TKO cells lines (Figure 2 and Table S1). These results suggest that pol V plays a particularly critical role in bypassing Z in the stronger replication blocking GZC sequence compared to the GZG sequence and that in the absence of pol V, the other Y-family pols such as pol II and pol IV also have a role in TLS of Z in this sequence context.

### Mutations resulting from bypass of Z in *E. coli*

**Figure 3:**
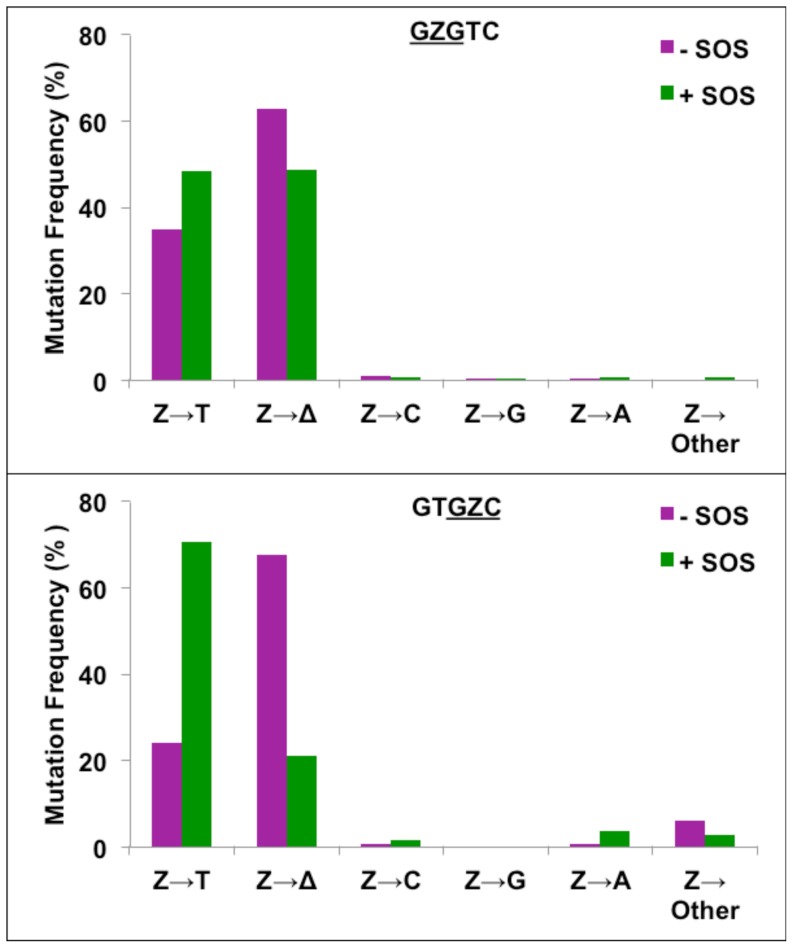
Mutations induced by Z in GZGTC and GTGZC sequence contexts in *E. coli* without (−) and with (+) SOS.

Analyses of the progeny plaques were carried out by oligonucleotide hybridization followed by DNA sequencing [37]. In the absence of SOS, the major type of mutation in both sequences was targeted one-base deletions (63% in GZG and 68% in GZC ) (Figure 3 and Table S2). Of the rest, most were Z→T (dAMP insertion), which occurred in 35% and 24% frequency, respectively, in GZG and GZC sequences. In the GZC sequence, multiple deletions were detected in 6% progeny, but they were undetectable in GZG sequence. dCMP, dTMP, and dGMP insertions were rare and occurred in <1% frequency. In the GZC sequence, Z→T increased from 24% in uninduced cells to 71% in SOS-induced cells, and one-base deletions dropped to 21%. In the GZG sequence, however, Z→T events increased to 49%, and one-base deletions also occurred at approximately 49% frequency. SOS had relatively minor effect on the other types of mutations. It is worth noting that significant differences in the TLS efficiency and nucleotide incorporation pattern opposite the AP-site depending on the DNA sequence context were reported by others as well [11], [19], and as with these studies, we cannot provide a rationale for the observed differences in TLS between the GZG and GZC sites.

**Figure 4:**
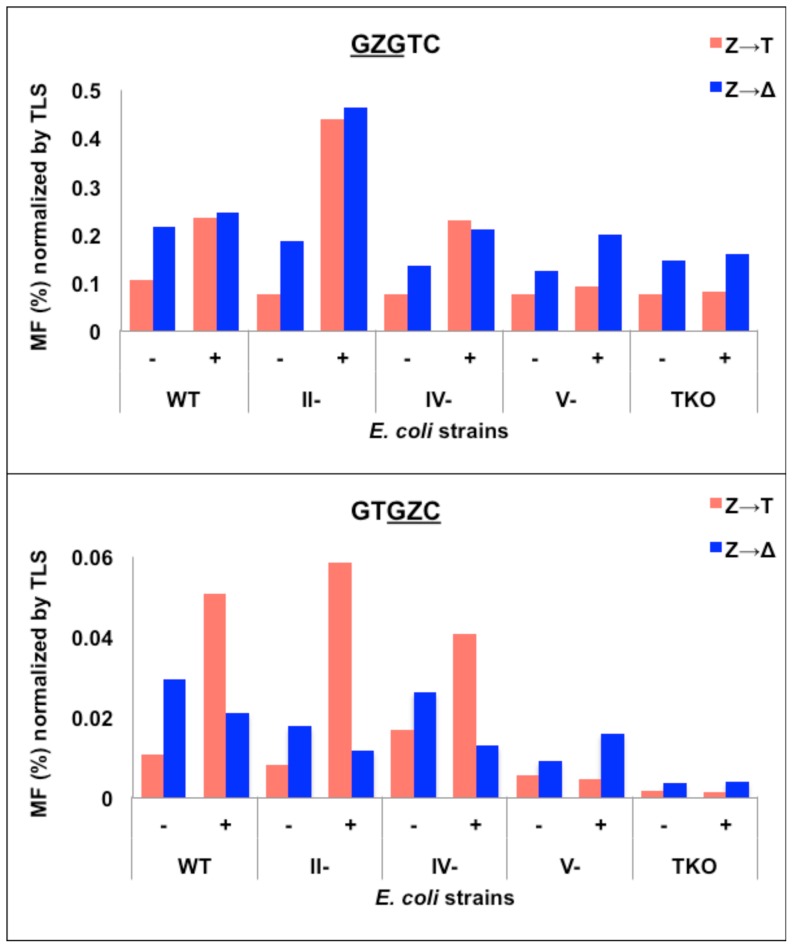
A comparison of the frequency of Z→T versus targeted Z deletion (i.e., Z→Δ) normalized by TLS (i.e., % MF multiplied with TLS frequency in hundredths) for GZGTC and GTGZC constructs in wild type and pol II-, pol IV-, pol V-, and TKO *E. coli* strains without (−) and with (+) SOS.

Since 92% and 98%, respectively, of the progeny in the GZC and GZG construct gave Z→T mutations and one-base deletions, for the polymerase-knockout strains, we only focused on these two types of mutations. In Figure 4, we report percent MF normalized by TLS to display both the relative proportion of the two main mutations combined with the bypass efficiency (whereas the % MF only is shown in Figure S1). As shown in Figure 4, Tables S3 and S4, in pol II- or pol IV-deficient cells, there were only minor changes in the relative proportion of the Z→T mutations or one-base deletions in GZG sequence. However, there were significant changes in the relative proportion of these two events in the GZC sequence. Without SOS, Z→T mutations increased from 24% in the wild type strain to 32% and 39%, respectively, in the pol II- and pol IV-deficient strains. With SOS, Z→T mutations increased from 71% in the wild type strain to 83% and 76%, respectively, in the pol II- and pol IV-deficient strains. In uninduced pol V-deficient strain also (Figure 4 and Table S5), Z→T mutations occurred at a higher frequency (38% in both sequences) than in the wild type cells (24% and 35%, respectively, in GZC and GZG sequence). In contrast, in both sequence contexts, the relative proportion of Z→T mutations dropped with SOS in pol V-deficient and TKO cells, except for GZG in TKO strain, where it remained approximately the same (Figure 4, Tables S5 and S6). These results indicate the major function of pol V is to insert dAMP opposite AP-sites, particularly with SOS when this pol is present in substantial concentration. But our results also suggest that in addition to the TLS pols, replicative pols are able to bypass AP-sites. Furthermore, substantial dAMP insertion occurred in the absence of pol V as well as other TLS pols, which suggests that replicative pols are able to insert dAMP across AP-sites. While the results of this study show pol V's primary role is to insert dAMP opposite AP-sites, it does not point to a specific pol for the one-base deletions.

### TLS of AP-site in human cells

**Figure 5:**
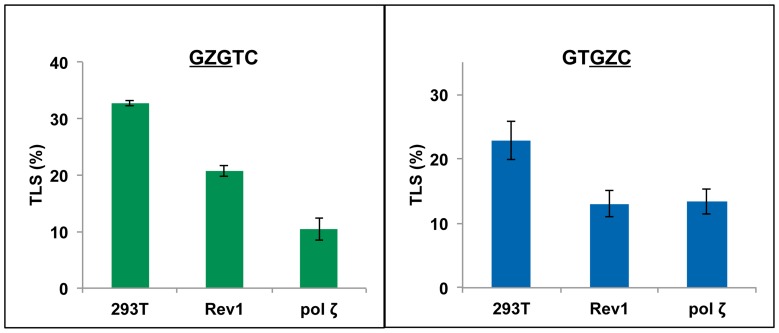
Effects of siRNA knockdowns of pol ζ and Rev1 on the extent of replicative bypass of Z for GZGTC and GTGZC constructs. Percent TLS in the pol knockdowns was measured using an internal control of unmodified plasmid containing a different sequence near the lesion site. When control siRNA was used, the % bypass remained the same as in HEK 293T cells.

To determine the magnitude of TLS in HEK 293T cells, we have mixed 2∶1 ratio of the AP-site construct and unmodified plasmid that contained a different sequence at the Z-containing 12-mer region. The unmodified plasmid was used as an internal control. The percentages of the colonies originating from the lesion-containing construct relative to the unmodified plasmid, reflecting the percentage of TLS, were determined by oligonucleotide hybridization. In stark contrast to *E. coli* cells, which gave less than 1% progeny for the Z-containing construct even with SOS, the frequency of TLS in HEK 293T cells was 23% and 33%, respectively, for the GZC and GZG sequence contexts (Figure 5 and Table S7). Even though Z in the GZC sequence posed a stronger replication block relative to GZG sequence in both *E. coli* and human cells, the abundance of TLS pols in the latter resulted in 2 to 3 order of magnitude more efficient TLS.

### AP-site mutagenesis in HEK 293T cells

**Figure 6:**
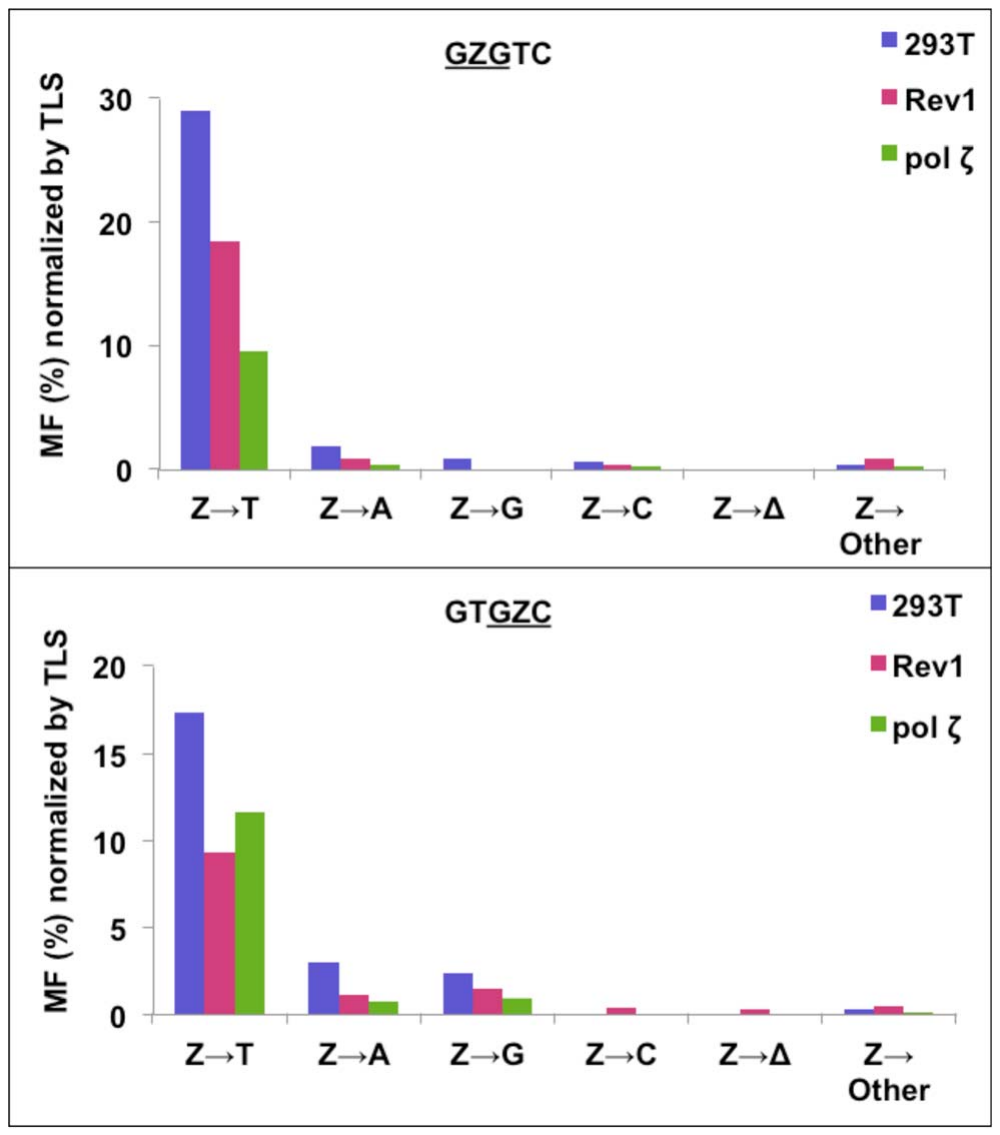
Percent mutations induced by Z in GZGTC and GTGZC sequence contexts normalized by TLS (i.e., % MF multiplied with TLS frequency in hundredths) for GZGTC and GTGZC constructs in HEK 293T cells without or with siRNA knockdowns of pol ζ and Rev1.

Figure 6 shows the percent MF normalized by TLS to display the relative proportions of various types of mutations combined with the bypass efficiencies in HEK 293T cells. Unlike the progeny from *E. coli* cells, the TLS of Z more rigidly followed the ‘A-rule.’ In the GZG and GZC sequence contexts, 89% and 76% Z→T, respectively, were detected (Figure 6, Figure S2 and Table S8). Moreover, unlike in *E. coli*, targeted one-base deletions were undetectable in both sequence contexts. The lesion in GZG and GZC sequences, respectively, yielded 6% and 13% Z→A, showing that dTMP insertion opposite Z was the second most prevalent event. dCMP insertion opposite the AP-site occurred at 2.5% and 10% frequency, respectively, in the GZG and GZC sequence. Of all the nucleotides inserted opposite the AP-site, dGMP was least favored, which occurred at 2% frequency in GZG sequence, but it was undetectable in the GZC sequence.

### Contribution of pol ζ and Rev1 in TLS of AP-site

**Figure 7:**
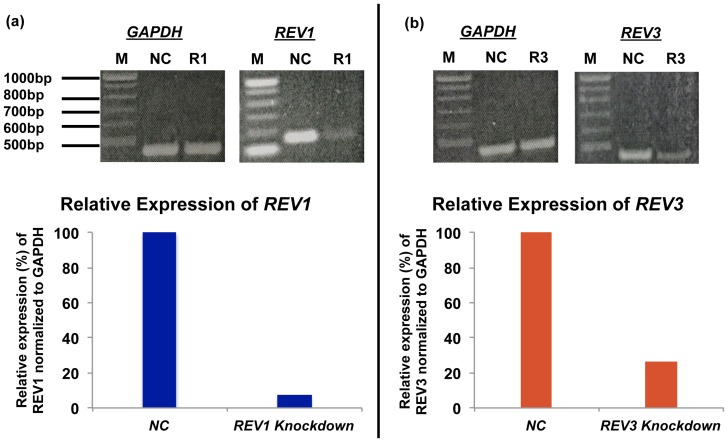
Representative gel images of siRNA knockdown of TLS pols in HEK293T cells. RT-PCR shows the efficiency of inhibition of the TLS pols. M, the DNA size marker; NC, negative control siRNA. Panel (a) shows the RT-PCR of Rev1 as R1; Panel (b) shows the same for Rev3, the catalytic subunit of pol ζ (R3). In each case, as a negative control of RT-PCR, the effects of siRNA were also inspected on glyceraldehyde-3-phosphate dehydrogenase (GAPDH) mRNA expression. The mRNA expressions of Rev1 and Rev3 of pol ζ relative to negative control (NC) are shown in the bar graph on the bottom.

In yeast, pol ζ was found essential for AP-site bypass [13]. It was also determined that Rev1 can proficiently insert a C opposite an AP-site, but it is unable to extend the replication product from there, whereas pol η is highly inefficient at both the insertion and extension steps of AP-site bypass [13], [28], [39]. To investigate the roles of pol ζ and Rev1 in AP-site bypass in HEK 293T cells, we employed siRNA knockdown approach to constrain the expression of these pols. The extent of siRNA knockdown was determined by RT-PCR (Figure 7) and by Western blotting analysis. For each pol, the knockdown was at least 75% efficient. When the cells were treated with a control siRNA, the extent of TLS of AP-site remained the same. By contrast, in the GZC sequence, knockdown of either pol ζ or Rev1 resulted in more than 40% reduction in TLS (Figure 5 and Table S7). In the GZG sequence, though, 36% and 69% reduction in TLS occurred in the Rev1 and pol ζ knockdown cells, respectively (Figure 5 and Table S7). Therefore, similar to the result in yeast [13], in human cells, pol ζ plays a key role; yet, it does not appear to be indispensable for AP-site bypass, because 30–57% bypass occurred in the absence of pol ζ. Whether the remaining low concentration of the enzyme in pol ζ knockdown cells is enough to carry out this level of TLS is a question we cannot address at this time. Rev1 also is important for TLS in human cells, but it does not insert dCMP opposite the AP-site. Rev1's likely role in AP-site bypass is the scaffolding function of the C-terminal domain that interacts with the Rev1-interacting region of other pols, including the Rev7 subunit of pol ζ [40], [41]. The type of mutations in the Rev1 and pol ζ knockdown cells, however, did not change appreciably from that in HEK 293T cells (Figure 6, Figure S2 and Table S8).

## Discussion

Analysis of bypass efficiency in *E. coli* indicated that TLS of AP-site is a particularly rare event and that pol V is required for 60–70% of the bypass in the GZC sequence, but it plays a less critical role in the GZG sequence. *In vitro* studies have shown that pol V can insert dAMP opposite an AP-site much more proficiently than pol III or pol IV [22], which was also indicated in our *in vivo* experiments. Thermodynamic studies have established that an A opposite the AP-site is enthalpically more favorable than a T [42], but it is likely that a pol can override thermodynamic considerations in the active site of the enzyme. In the absence of SOS in the current study, the major bypass involved one-base deletions. The mechanism of one-base deletion is not clear. Although dCMP insertion opposite the AP-site followed by slippage in GZN sequence to pair with the 5′G can be postulated, it cannot account for the lack of slippage when dAMP is incorporated opposite AP-site in TZG sequence used in another study [19]. Perhaps the GC pair can stabilize the slippage more efficiently than the AT pair. The role of pol II, pol IV, or both in one-base deletion is likely, but more experiments are needed to rationalize the context effects. With SOS, the full-length products were increased to 49% in GZG and 71% in GZC sequence, with predominantly dAMP inserted opposite the AP-site. In the GZG sequence, one-base deletions occurred at the same frequency as dAMP insertions. Livneh and coworkers have analyzed TLS of AP-site in a GZG sequence, but except for the immediate neighbors, the sequence was different from this study [43]. Furthermore, the lesion was located in the single-stranded gap region of a gapped duplex plasmid, and TLS gave 76% dAMP insertions opposite the AP-site and 21% targeted one-base deletions with SOS. While both pol IV and pol V can bypass Z *in vitro* [43], [44], pol V is much more efficient than pol IV [22], and pol V and not pol IV was suggested to play a major role *in vivo* [43]. Besides, suggestions have been made that TLS by pol V results in base substitutions, whereas deletions are more likely to result from bypass by pol IV or even pol III, although the latter is much less efficient in bypassing AP-sites [22], [43]. In our study a clear-cut role of pol IV in deletion was not identified.

TLS studies on AP-site from different laboratories have shown that less than 1% TLS in *E. coli* occur in the absence of SOS, which increased 2- to 10-fold with SOS, whereas about 6% TLS takes place in yeast cells in double-stranded DNA [11], [13], [19]. In contrast, in the single-stranded gap region of a gapped duplex plasmid, 20–90% TLS of AP-site have been reported to occur in human cells [17]. Our comparative study in *E. coli* and human cells using the same sequence context is consistent with these reports. In contrast to *E. coli*, in which less than 1% TLS occurred, in HEK 293T cells we observed 23% and 33% TLS in the GZC and GZG sequence contexts. As in *E. coli*, GZC site was a stronger replication block than GZG , but much higher TLS in both sites suggests that the pols in human cells are more proficient in bypassing AP-sites. dAMP insertion opposite the AP-site was the dominant incidence in both sites with >75% Z→T substitutions. The second and third most prevalent events were dTMP and dCMP insertions, respectively, whereas dGMP insertion occurred at a very low frequency. One-base deletion, the major outcome noted in *E. coli*, was undetectable. This is noteworthy because *in vitro* studies on AP-site templates show that pol β and pol λ can promote template slippage to generate −1 frameshifts [45]. Our result, therefore, implies that these X-family pols are not involved in TLS of AP-site during replication in HEK 293T cells, although they might be important for re-synthesis step of repair pathways. Evidently, the bypass polymerases in mammalian cells can avoid the deleterious consequence of frameshift mutations, which parallels our earlier observation in simian kidney cells [32]. Using a gapped plasmid vector system in human adenocarcinoma H1299 cells, insertion of dAMP opposite Z was reported [17], as we have observed in HEK 293T cells. We also determined that pol ζ is a critical bypass pol for TLS of AP-site in human cells, since 44–67% drop in TLS have occurred in pol ζ knockdown cells. Likewise, Rev1 also is important for AP-site bypass, as shown by 36–43% reduction in TLS in Rev1 knockdown cells. Since Rev1 does not serve as a deoxycytidyl transferase in TLS of AP-sites, it probably acts as a structural element for another pol such as pol ζ. But, unlike in yeast [13], neither pol ζ nor Rev1 was deemed indispensable in human cells. However, future experiments with pol ζ- and Rev1-knockout cells will be required to address this question with certainty. It was suggested that AP-site bypass in human cells requires at least one of the replicative pols, α, δ, or ε [17]. Although we did not investigate the roles of the replicative pols in AP-site bypass in human cells, our results are not inconsistent with this notion.

## Supporting Information

Figure S1 **A comparison of the frequency of Z→T versus targeted Z deletion (i.e., Z→Δ) for the GZGTC and GTGZC constructs in wild type and pol II-, pol IV-, pol V-, and TKO** ***E. coli*** **strains without (−) and with (+) SOS.**
(TIF)

Figure S2 **Percent mutations induced by Z in GZGTC and GTGZC sequence contexts for the GZGTC and GTGZC constructs in HEK 293T cells without or with siRNA knockdowns of pol ζ and Rev1.**
(TIF)

Table S1 **Viability of abasic site in** ***E. coli*** **strains.**
(DOCX)

Table S2 **Mutational frequency in wild type** ***E. coli*** **cells.**
(DOCX)

Table S3 **Mutation frequency in pol II- deficient** ***E. coli*** **strain.**
(DOCX)

Table S4 **Mutation frequency in pol IV- deficient** ***E. coli*** **strain.**
(DOCX)

Table S5 **Mutation frequency in pol V- deficient** ***E. coli*** **strain.**
(DOCX)

Table S6 **Mutation frequency in triple knockout** ***E. coli*** **strain.**
(DOCX)

Table S7 **TLS % in different polymerase knockdown HEK 293T cells.**
(DOCX)

Table S8 **Mutation frequency in TLS polymerase knockdown HEK 293T cells.**
(DOCX)
