# Correction: Replicative Bypass of Abasic Site in *Escherichia coli* and Human Cells: Similarities and Differences

**DOI:** 10.1371/journal.pone.0112506

**Published:** 2014-10-28

**Authors:** The PLOS ONE Staff

## Notice of Republication

This article was republished on October 16, 2014, to correct the order and numbering of Figures 5-7, as well as one in-text figure citation. Please download this article again to view the correct version. The originally published, uncorrected article and the republished, corrected article are provided here for reference.

## Supporting Information

File S1 Originally published, uncorrected article.
(PDF)

File S2 Republished, corrected article.
(PDF)
