# Supplementary figures and images for: Replicative Bypass of Abasic Site in Escherichia coli and Human Cells: Similarities and Differences
Source: PLoS One. 2014 Sep 16;9(9):e107915. doi: 10.1371/journal.pone.0107915 (PMC4167244; doi:10.1371/journal.pone.0107915)

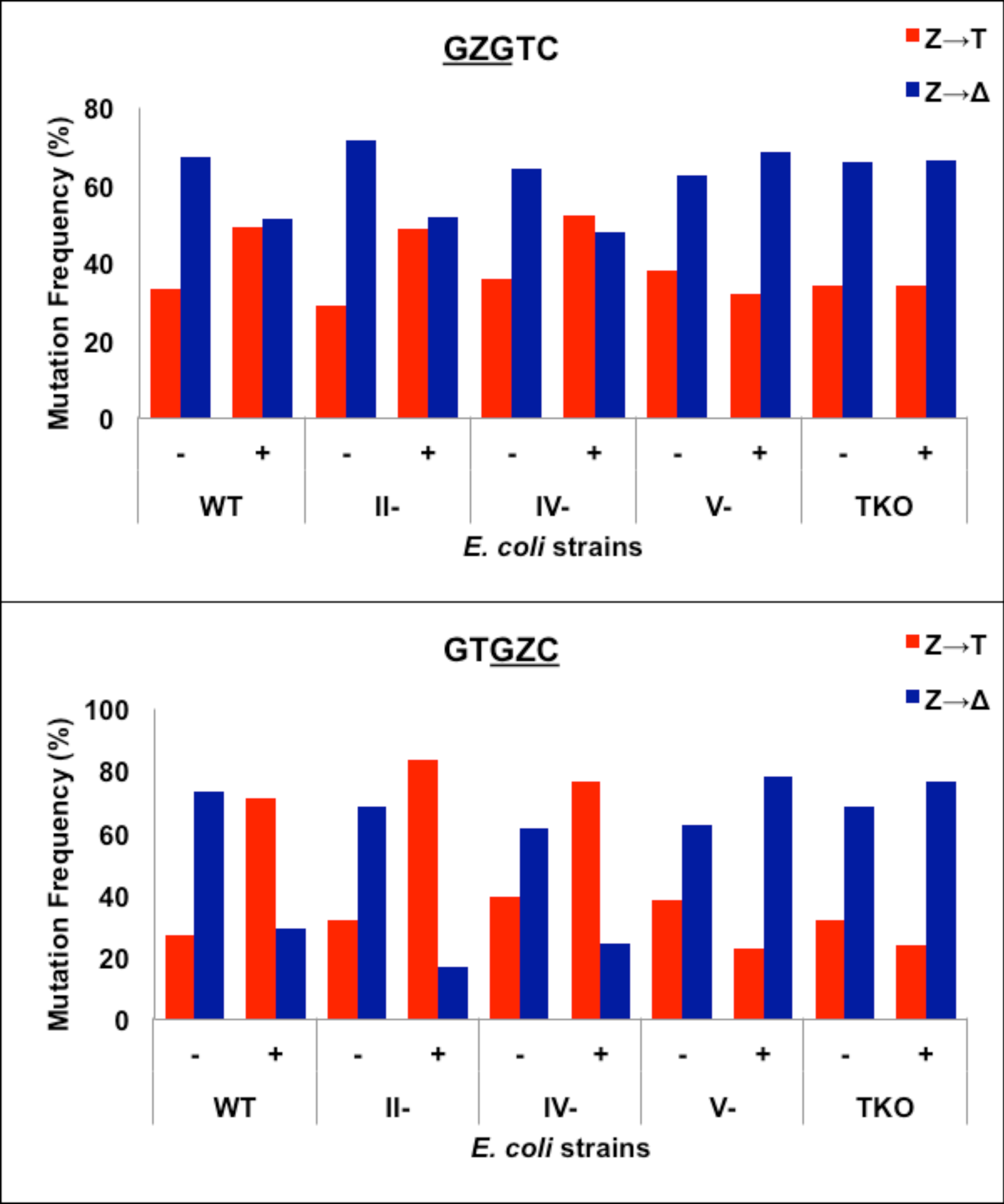

Supplement: Figure S1 — A comparison of the frequency of Z→T versus targeted Z deletion (i.e., Z→Δ) for the GZGTC and GTGZC constructs in wild type and pol II-, pol IV-, pol V-, and TKO E. coli strains without (−) and with (+) SOS. (TIF) [file pone.0107915.s001.tif]

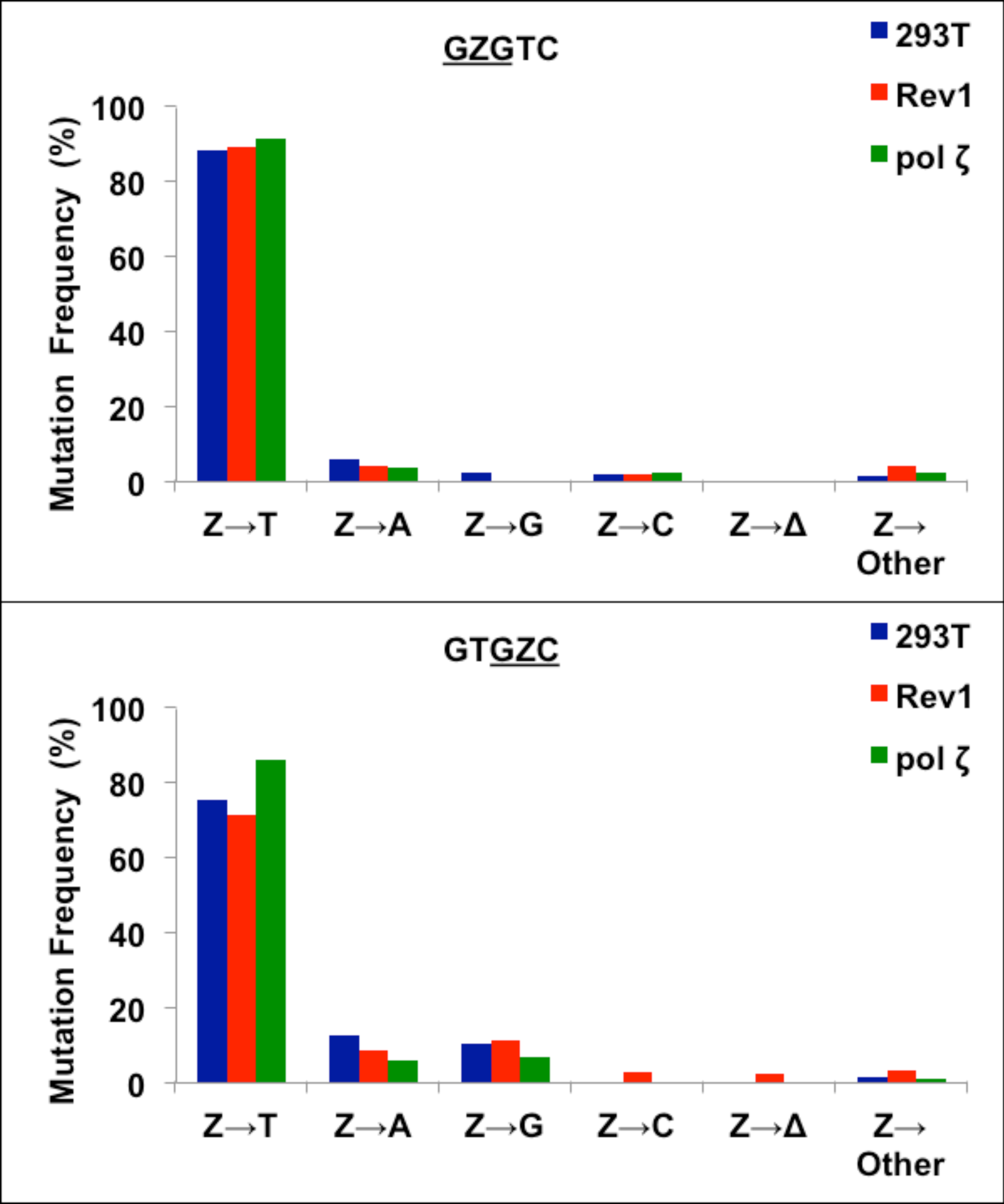

Supplement: Figure S2 — Percent mutations induced by Z in GZGTC and GTGZC sequence contexts for the GZGTC and GTGZC constructs in HEK 293T cells without or with siRNA knockdowns of pol ζ and Rev1. (TIF) [file pone.0107915.s002.tif]
