# Supplementary material for: Replicative Bypass of Abasic Site in Escherichia coli and Human Cells: Similarities and Differences
Source: PLoS One. 2014 Sep 16;9(9):e107915. doi: 10.1371/journal.pone.0107915 (PMC4167244; doi:10.1371/journal.pone.0107915)
Supplement: Table S1 — Viability of abasic site in E. coli strains. (DOCX) [file pone.0107915.s003.docx]

**Table S1.** Viability of abasic site in *E. coli* strains

| **Strain** | **SOS** | **GZGTC** | | **GTGZC** | |
| --- | --- | --- | --- | --- | --- |
| WT | - | 0.3 | ± 0.2 | 0.04 | ± 0.01 |
|  | + | 0.48 | ± 0.06 | 0.07 | ± 0.002 |
|  |  |  |  |  |  |
| pol II- | - | 0.26 | ± 0.03 | 0.03 | ±0.004 |
|  | + | 0.9 | ± 0.1 | 0.07 | ±0.04 |
|  |  |  |  |  |  |
| pol IV- | - | 0.21 | ±0.06 | 0.04 | ±0.01 |
|  | + | 0.44 | ± 0.01 | 0.05 | ± 0.01 |
|  |  |  |  |  |  |
| pol V- | - | 0.20 | ±0.02 | 0.01 | ± 0.01 |
|  | + | 0.29 | ± 0.09 | 0.02 | ± 0.009 |
|  |  |  |  |  |  |
| TKO | - | 0.22 | ±0.01 | 0.005 | ±0.002 |
|  | + | 0.24 | ±0.07 | 0.005 | ±0.006 |
|  |  |  |  |  |  |
