# Supplementary material for: Replicative Bypass of Abasic Site in Escherichia coli and Human Cells: Similarities and Differences
Source: PLoS One. 2014 Sep 16;9(9):e107915. doi: 10.1371/journal.pone.0107915 (PMC4167244; doi:10.1371/journal.pone.0107915)
Supplement: Table S2 — Mutational frequency in wild type E. coli cells. (DOCX) [file pone.0107915.s004.docx]

**Table S2.** Mutational frequency in wild type *E. coli* cells^†^

| Strain | Lesion | SOS | Trial | Total plaques screened | Z →T (%) | | Z →Δ (%) | | Z →C (%) | | Z →G (%) | | Z→A (%) | | Z→ Other (%) | |
| --- | --- | --- | --- | --- | --- | --- | --- | --- | --- | --- | --- | --- | --- | --- | --- | --- |
| WT | **GZGTC** | - | 1 | 85 | 31^a^ | (37) | 52 | (61) | 2 | (2) | 0 | (0.0) | 0 | (0.0) | 0 | (0.0) |
|  |  |  | 2 | 101 | 34^b^ | (34) | 65 | (64) | 0 | (0.0) | 1 | (1) | 1^c^ | (1) | 0 | (0.0) |
|  |  |  | **Total** | **186** | **65** | **(35)** | **117** | **(63)** | **2** | **(1)** | **1** | **(0.5)** | **1** | **(0.5)** | **0** | **(0.0)** |
|  |  |  |  |  |  |  |  |  |  |  |  |  |  |  |  |  |
|  |  | + | 1 | 156 | 74 | (47) | 77 | (49) | 1 | (1) | 1^d^ | (1) | 1 | (1) | 2^e^ | (1) |
|  |  |  | 2 | 124 | 62 | (50) | 60 | (48) | 1 | (1) | 0 | (0.0) | 1 | (1) | 0 | (0.0) |
|  |  |  | **Total** | **280** | **136** | **(49)** | **133** | **(49)** | **2** | **(0.7)** | **1** | **(0.4)** | **2** | **(0.7)** | **2** | **(0.7)** |
|  |  |  |  |  |  |  |  |  |  |  |  |  |  |  |  |  |
|  | **GTGZC** | - | 1 | 54 | 11^f^ | (20) | 41^g^ | (76) | 0 | (0.0) | 0 | (0.0) | 0 | (0.0) | 2^h^ | (4) |
|  |  |  | 2 | 57 | 16 | (28) | 34^i^ | (60) | 1 | (2) | 0 | (0.0) | 1^j^ | (2) | 5^h,k,l^ | (9) |
|  |  |  | **Total** | **107** | **27** | **(24)** | **74** | **(68)** | **1** | (1) | **0** | **(0.0)** | **1** | (1) | **7** | **(6)** |
|  |  |  |  |  |  |  |  |  |  |  |  |  |  |  |  |  |
|  |  | + | 1 | 71 | 51^m^ | (72) | 13 | (19) | 2 | (3) | 0 | (0.0) | 3^j,n^ | (4) | 2^k,o^ | (3) |
|  |  |  | 2 | 38 | 26 | (68) | 10 | (26) | 0 | (0.0) | 0 | (0.0) | 1^j^ | (3) | 1^p^ | (3) |
|  |  |  | **Total** | **109** | **77** | **(71)** | **23** | **(21)** | **2** | **(2)** | **0** | **(0.0)** | **4** | **(4)** | **3** | **(3)** |
|  |  |  |  |  |  |  |  |  |  |  |  |  |  |  |  |  |

**^†^**The superscript indicates one or more mutants containing mutation elsewhere shown below and the number in parenthesis shows the number of events detected.

(a) TGC AGT TTC CGT (1), (b) CGC AGT GTC CGT (1), (c) TGC AGA ATC CGT (1), (d) TAG AAG GTC ACG (1), (e) TG _ _ _ _ _ _ _ AGC, _ _ _ _ _ _ _ TG AGC (1),(f) TGC AG _ GTC AGC (1), TGC A _ _ GTC AGC (1), TGC A _ _ CTC AGC (1),( g) TGC AG _ G _ C AGC (1), T_ G AGT G_C AGC (1), (h)TGC AG _ _ _ _ _ _ _ (2 in expt 1 and 2 in expt 2), (i) TGC AGT _ _C AGC, TGC AGT G_ C CGC (1),(j) TGC AGT _ AC AGC (2 in expt 1 and 1 in expt 2), (k) _ _ _ _ _ _ _ _ _ _ GC (1), (l)TGC _ _ _ _ _ _ AGC, TG_ _ _ _ _ _ C AGC (1) , (m) TGC AGA GTC AGC (1), (n) _ _ _ _ _ _ _ AC AGC (1),(o) TGC A _ _ _ _ C AGC (1), (p) _ _ _ _ _ _ _ _ _ C AGC (1)
