# Supplementary material for: Replicative Bypass of Abasic Site in Escherichia coli and Human Cells: Similarities and Differences
Source: PLoS One. 2014 Sep 16;9(9):e107915. doi: 10.1371/journal.pone.0107915 (PMC4167244; doi:10.1371/journal.pone.0107915)
Supplement: Table S3 — Mutation frequency in pol II- deficient E. coli strain. (DOCX) [file pone.0107915.s005.docx]

**Table S3.** Mutation frequency in pol II- deficient *E. coli* strain

| Strain | Lesion | SOS | Trial | Total plaques signals | Z→T (%) | | Z→Δ(%) | |
| --- | --- | --- | --- | --- | --- | --- | --- | --- |
| pol II- | **GZGTC** | - |  |  |  |  |  |  |
|  |  |  | 1 | 86 | 27 | (31) | 59 | (69) |
|  |  |  | 2 | 268 | 75 | (28) | 193 | (72) |
|  |  |  | **Total** | **354** | **102** | **(29)** | **252** | **(71)** |
|  |  |  |  |  |  |  |  |  |
|  |  | + | 1 | 211 | 99 | (47) | 112 | (53) |
|  |  |  | 2 | 63 | 29 | (46) | 34 | (54) |
|  |  |  | 3 | 117 | 62 | (53) | 55 | (47) |
|  |  |  | **Total** | **391** | **190** | **(49)** | **201** | **(51)** |
|  |  |  |  |  |  |  |  |  |
|  | **GTGZC** |  |  |  |  |  |  |  |
|  |  | - | 1 | 25 | 9 | (36) | 16 | (64) |
|  |  |  | 2 | 13 | 3 | (23) | 10 | (77) |
|  |  |  | **Total** | **38** | **12** | **(32)** | **26** | **(68)** |
|  |  |  |  |  |  |  |  |  |
|  |  | + | 1 | 45 | 33 | (73) | 12 | (27) |
|  |  |  | 2 | 85 | 78 | (92) | 7 | (8) |
|  |  |  | 3 | 56 | 44 | (79) | 12 | (21) |
|  |  |  | **Total** | **186** | **155** | **(83)** | **31** | **(17)** |
|  |  |  |  |  |  |  |  |  |
