# Supplementary material for: Replicative Bypass of Abasic Site in Escherichia coli and Human Cells: Similarities and Differences
Source: PLoS One. 2014 Sep 16;9(9):e107915. doi: 10.1371/journal.pone.0107915 (PMC4167244; doi:10.1371/journal.pone.0107915)
Supplement: Table S4 — Mutation frequency in pol IV- deficient E. coli strain. (DOCX) [file pone.0107915.s006.docx]

**Table S4.** Mutation frequency in pol IV- deficient *E. coli* strain

| Strain | Lesion | SOS | Trial | Total plaques screened | Z→T (%) | | Z→Δ (%) | |
| --- | --- | --- | --- | --- | --- | --- | --- | --- |
| pol IV- | **GZGTC** | - | 1 | 115 | 39 | (34) | 76 | (66) |
|  |  |  | 2 | 89 | 34 | (38) | 55 | (62) |
|  |  |  | **Total** | **204** | **73** | **(36)** | **131** | **(64)** |
|  |  |  |  |  |  |  |  |  |
|  |  | + | 1 | 175 | 87 | (50) | 88 | (50) |
|  |  |  | 2 | 190 | 103 | (54) | 87 | (46) |
|  |  |  | **Total** | **365** | **190** | **(52)** | **175** | **(48)** |
|  |  |  |  |  |  |  |  |  |
|  | **GTGZC** | - | 1 | 30 | 12 | (40) | 18 | (60) |
|  |  |  | 2 | 29 | 11 | (38) | 18 | (62) |
|  |  |  | **Total** | **59** | **23** | **(39)** | **36** | **(61)** |
|  |  |  |  |  |  |  |  |  |
|  |  | + | 1 | 80 | 60 | (75) | 20 | (25) |
|  |  |  | 2 | 37 | 29 | (78) | 8 | (22) |
|  |  |  | **Total** | **117** | **89** | **(76)** | **28** | **(24)** |
|  |  |  |  |  |  |  |  |  |
