# Supplementary material for: Replicative Bypass of Abasic Site in Escherichia coli and Human Cells: Similarities and Differences
Source: PLoS One. 2014 Sep 16;9(9):e107915. doi: 10.1371/journal.pone.0107915 (PMC4167244; doi:10.1371/journal.pone.0107915)
Supplement: Table S5 — Mutation frequency in pol V- deficient E. coli strain. (DOCX) [file pone.0107915.s007.docx]

**Table S5.** Mutation frequency in pol V- deficient *E. coli* strain

| Strain | Lesion | SOS | Trial | Total plaques screened | Z→T | (%) | Z→Δ | (%) |
| --- | --- | --- | --- | --- | --- | --- | --- | --- |
| pol V- | **GZGTC** | - | 1 | 77 | 29 | (38) | 48 | (62) |
|  |  |  | 2 | 146 | 53 | (36) | 93 | (64) |
|  |  |  | 3 | 225 | 87 | (39) | 138 | (61) |
|  |  |  | **Total** | **448** | **169** | **(38)** | **279** | **(62)** |
|  |  | + |  |  |  |  |  |  |
|  |  |  | 1 | 34 | 10 | (29) | 24 | (71) |
|  |  |  | 2 | 236 | 78 | (33) | 158 | (67) |
|  |  |  | 3 | 117 | 35 | (30) | 82 | (70) |
|  |  |  | **Total** | **387** | **123** | **(32)** | **264** | **(68)** |
|  |  |  |  |  |  |  |  |  |
|  | **GTGZC** | - | 1 | 18 | 5 | (28) | 13 | (72) |
|  |  |  | 2 | 40 | 17 | (43) | 23 | (58) |
|  |  |  | **Total** | **58** | **22** | **(38)** | **36** | **(62)** |
|  |  |  |  |  |  |  |  |  |
|  |  | + | 1 | 20 | 4 | (20) | 16 | (80) |
|  |  |  | 2 | 59 | 12 | (20) | 47 | (80) |
|  |  |  | 3 | 87 | 21 | (24) | 66 | (76) |
|  |  |  | **Total** | **166** | **37** | **(22)** | **129** | **(78)** |
|  |  |  |  |  |  |  |  |  |
