# Supplementary material for: Replicative Bypass of Abasic Site in Escherichia coli and Human Cells: Similarities and Differences
Source: PLoS One. 2014 Sep 16;9(9):e107915. doi: 10.1371/journal.pone.0107915 (PMC4167244; doi:10.1371/journal.pone.0107915)
Supplement: Table S6 — Mutation frequency in triple knockout E. coli strain. (DOCX) [file pone.0107915.s008.docx]

**Table S6.** Mutation frequency in triple knockout *E. coli* strain

| Strain | Lesion | SOS | Trial | Total Plaques screened | Z→T (%) | | Z→Δ (%) | |
| --- | --- | --- | --- | --- | --- | --- | --- | --- |
|  |  |  |  |  |  |  |  |  |
| TKO | **GZGTC** | - | 1 | 137 | 48 | (35) | 89 | (65) |
|  |  |  | 2 | 178 | 59 | (33) | 119 | (67) |
|  |  |  | **Total** | **315** | **107** | **(34)** | **208** | **(66)** |
|  |  |  |  |  |  |  |  |  |
|  |  | + | 1 | 73 | 23 | (32) | 50 | (68) |
|  |  |  | 2 | 92 | 33 | (36) | 59 | (64) |
|  |  |  | **Total** | **165** | **56** | **(34)** | **109** | **(66)** |
|  |  |  |  |  |  |  |  |  |
|  | **GTGZC** | - | 1 | 11 | 3 | (27) | 8 | (73) |
|  |  |  | 2 | 58 | 19 | (33) | 39 | (67) |
|  |  |  | **Total** | **69** | **22** | **(32)** | **47** | **(68)** |
|  |  |  |  |  |  |  |  |  |
|  |  | + | 1 | 17 | 5 | (29) | 12 | (71) |
|  |  |  | 2 | 21 | 4 | (19) | 17 | (81) |
|  |  |  | **Total** | **38** | **9** | **(24)** | **29** | **(76)** |
