# Supplementary material for: Replicative Bypass of Abasic Site in Escherichia coli and Human Cells: Similarities and Differences
Source: PLoS One. 2014 Sep 16;9(9):e107915. doi: 10.1371/journal.pone.0107915 (PMC4167244; doi:10.1371/journal.pone.0107915)
Supplement: Table S7 — TLS % in different polymerase knockdown HEK 293T cells. (DOCX) [file pone.0107915.s009.docx]

**Table S7**. TLS % in different polymerase knockdown HEK 293T cells

| **GZGTC** | **Strain** | **Trial** | **TLS %** |
| --- | --- | --- | --- |
|  | 293T | 1 | 33 |
|  |  | 2 | 32 |
|  |  | **AVG** | **32.5±0.5** |
|  | Rev 1 | 1 | 22 |
|  |  | 2 | 20 |
|  |  | **AVG** | **21±1** |
|  | pol ζ | 1 | 13 |
|  |  | 2 | 8 |
|  |  | 3 | 12 |
|  |  | 4 | 8 |
|  |  | **AVG** | **10±2** |

| **GTGZC** | **Strain** | **Trial** |  | **TLS %** |
| --- | --- | --- | --- | --- |
|  | **293T** | **1** |  | **20** |
|  |  | **2** |  | **26** |
|  |  | **AVG** |  | **23±3** |
|  | **Rev 1** | **1** |  | **11** |
|  |  | **2** |  | **15** |
|  |  | **AVG** |  | **13±2** |
|  | **pol ζ** | **1** |  | **11** |
|  |  | **2** |  | **15** |
|  |  | **3** |  | **14** |
|  |  | **AVG** |  | **13±2** |
