# Supplementary material for: Replicative Bypass of Abasic Site in Escherichia coli and Human Cells: Similarities and Differences
Source: PLoS One. 2014 Sep 16;9(9):e107915. doi: 10.1371/journal.pone.0107915 (PMC4167244; doi:10.1371/journal.pone.0107915)
Supplement: Table S8 — Mutation frequency in TLS polymerase knockdown HEK 293T cells. (DOCX) [file pone.0107915.s010.docx]

**Table S8**. Mutation frequency in TLS polymerase knockdown HEK 293T cells**^†^**

**^†^**The superscript indicates one or more mutants containing mutation elsewhere shown below and the number in parenthesis shows the number of events detected.

| **Lesion** |  | **Trial** | **Total**  **Signals** | **Z →T** | **(%)** | **Z**→**A** | **(%)** | **Z**→**G** | **(%)** | **Z**→**C** | **(%)** | **Z**→**Del** | **(%)** | **Other** | **(%)** |
| --- | --- | --- | --- | --- | --- | --- | --- | --- | --- | --- | --- | --- | --- | --- | --- |
| **GZGTC** | **293T** | 1 | 43 | 37^a,b^ | (86) | 3 | (7) | 3^c^ | (7) | 0 | (0) | 0 | (0) | 0 | (0) |
|  |  | 2 | 61 | 54^d^ | (89) | 4 | (7) | 0 | (0) | 1 | (2) | 0 | (0) | 2^e^ | (3) |
|  |  | 3 | 53 | 48^f^ | (91) | 2 | (4) | 1 | (2) | 2 | (4) | 0 | (0) | 0 | (0) |
|  | **Total** |  | **157** | **139** | **(89)** | **9** | **(6)** | **4** | **(3)** | **3** | **(2)** | **0** | **(0)** | **2** | **(1)** |
|  |  |  |  |  |  |  |  |  |  |  |  |  |  |  |  |
|  | **Rev 1** | 1 | 41 | 37^b^ | (90) | 2 | (5) | 0 | (0) | 1 | (2) | 0 | (0) | 1^g^ | (2) |
|  |  | 2 | 53 | 47^a^ | (89) | 2 | (4) | 0 | (0) | 1 | (2) | 0 | (0) | 3^g^ | (6) |
|  | **Total** |  | **94** | **84** | **(89)** | **4** | **(4)** | **0** | **(0)** | **2** | (2) | **0** | **(0)** | **4** | **(4)** |
|  | **Pol ζ** | 1 | 82 | 74^b^ | (90) | 3 | (4) | 0 | (0) | 2 | (2) | 0 | (0) | 3^g^ | (4) |
|  |  | 2 | 46 | 43^b,h^ | (94) | 2 | (4) | 0 | (0) | 1 | (2) | 0 | (0) | 0 | (0) |
|  | **Total** |  | **128** | **117** | **(91)** | **5** | **(4)** | **0** | **(0)** | **3** | **(2)** | **0** | **(0)** | **3** | **(2)** |

(a) TGC CGT GTC AGC (1), (b) TGC ATT GTC AGC (1) (c) TGC GAG GTC AGC (2), (d) TGC _ _ T GTC AGC (1), (e)_ _ _ _ _ _ GTC AGC (1), (f) TGC GAT GTC AGC (2), (g) _ _ _ _ _ _ _ _ C AGC (1 in expt 1, 3 in expt 2 of Rev1; 3 in expt 1 of pol ζ ), (h) _ _ _ AGT GTC AGC (1)

| **Lesion** |  | **Trial** | **Total Signals** | **Z →T** | **(%)** | **Z**→**A** | **(%)** | **Z**→**G** | **(%)** | **Z**→**C** | **(%)** | **Z**→ **Del (%)** | | **Other** | **(%)** |
| --- | --- | --- | --- | --- | --- | --- | --- | --- | --- | --- | --- | --- | --- | --- | --- |
| **GTGZC** | **293T** | 1 | 27 | 20 | (74) | 4 | (15) | 3 | (11) | 0 | (0) | 0 | (0) | 0 | (0) |
|  |  | 2 | 51 | 39^i^ | (77) | 6 | (12) | 5 | (10) | 0 | (0) | 0 | (0) | 1^j^ | (2) |
|  | **Total** |  | **78** | **59** | (76) | **10** | **(13)** | **8** | **(10)** | **0** | **(0)** | **0** | **(0)** | **1** | **(1)** |
|  |  |  |  |  |  |  |  |  |  |  |  |  |  |  |  |
|  | **REV1** | 1 | 78 | 56^i^ | (72) | 9 | (12) | 10 | (13) | 2^k^ | (3) | 0 | (0) | 1^l^ | (1) |
|  |  | 2 | 39 | 25^i^ | (64) | 4 | (10) | 6 | (15) | 1^m^ | (3) | 3 | (8) | 0 | (0) |
|  |  | 3 | 59 | 45^i,n^ | (76) | 2 | (3) | 4 | (7) | 2 | (3) | 1 | (2) | 5^l,o^ | (9) |
|  | **Total** |  | **176** | **126** | **(72)** | **15** | **(9)** | **20** | **(11)** | **5** | **(3)** | **4** | **(2)** | **6** | **(3)** |
|  | **pol ζ** | 1 | 54 | 48 | (89) | 3 | (6) | 3 | (6) | 0 | (0) | 0 | (0) | 0 | (0) |
|  |  | 2 | 34 | 28 | (82) | 2^p^ | (6) | 3^q^ | (9) | 0 | (0) | 0 | (0) | 1^o^ | (3) |
|  | **Total** |  | **88** | **76** | **(86)** | **5** | **(6)** | **6** | **(7)** | **0** | **(0)** | **0** | **(0)** | **1** | **(1)** |
|  |  |  |  |  |  |  |  |  |  |  |  |  |  |  |  |

**^†^**The superscript indicates one or more mutants containing mutation elsewhere shown below and the number in parenthesis shows the number of events detected.

(i) TGC AAT GTC AGC (1 in 293T, 1 each in expt 1 & 3 and 2 in expt 2 of Rev1), (j) TG - - - - G- C AGC (1) ,(k) TGC AG- CCC AGC (1) ,(l) - - - - - - - - - C AGC (4), (m) TGC AGT ACC AGC (1), (n) _ _ _ _ _ _ TT C ACG(1), TGC TGT GTC AGC(1), _ _ _ AGT GTC AGC (1), (o) T_ _ _ _ _ _ _ _ AGC (1) , (p) _ _ _ GAT GAC AGC (1), (q) TGC AAT GGC AGC (1)
